# From Nanobots to Neural Networks: Multifaceted Revolution of Artificial Intelligence in Surgical Medicine and Therapeutics

**DOI:** 10.7759/cureus.49082

**Published:** 2023-11-19

**Authors:** Han Grezenko, Lara Alsadoun, Ayesha Farrukh, Abdur Rehman, Abdullah Shehryar, Eemaz Nathaniel, Maryam Affaf, Mohammed Khaleel I KH Almadhoun, Maria Quinn

**Affiliations:** 1 Translational Neuroscience, Barrow Neurological Institute, Phoenix, USA; 2 Plastic Surgery, Chelsea and Westminster Hospital, London, GBR; 3 Family Medicine, Rawalpindi Medical University, Rawalpindi, PAK; 4 Surgery, Mayo Hospital, Lahore, PAK; 5 Internal Medicine, Allama Iqbal Medical College, Lahore, PAK; 6 Research, Rehman Medical Institute, Peshawar, PAK; 7 Internal Medicine, Women's Medical and Dental College, Abbotabad, PAK; 8 Medicine and Surgery, Mutah University, Karak, JOR; 9 Internal Medicine, Jinnah Hospital, Lahore, PAK

**Keywords:** cybersecurity, ethical ai, data usage, patient consent, data breaches, patient privacy, data security, healthcare, ai integration, artificial intelligence

## Abstract

This comprehensive exploration unveils the transformative potential of Artificial Intelligence (AI) within medicine and surgery. Through a meticulous journey, we examine AI's current applications in healthcare, including medical diagnostics, surgical procedures, and advanced therapeutics. Delving into the theoretical foundations of AI, encompassing machine learning, deep learning, and Natural Language Processing (NLP), we illuminate the critical underpinnings supporting AI's integration into healthcare.

Highlighting the symbiotic relationship between humans and machines, we emphasize how AI augments clinical capabilities without supplanting the irreplaceable human touch in healthcare delivery. Also, we'd like to briefly mention critical findings and takeaways they can expect to encounter in the article. A thoughtful analysis of the economic, societal, and ethical implications of AI's integration into healthcare underscores our commitment to addressing critical issues, such as data privacy, algorithmic transparency, and equitable access to AI-driven healthcare services.

As we contemplate the future landscape, we project an exciting vista where more sophisticated AI algorithms and real-time surgical visualizations redefine the boundaries of medical achievement. While acknowledging the limitations of the present research, we shed light on AI's pivotal role in enhancing patient engagement, education, and data security within the burgeoning realm of AI-driven healthcare.

## Introduction and background

The fusion of Artificial Intelligence (AI) with the world of medicine, especially within the field of surgery, marks a significant turning point poised to reshape the healthcare landscape. With its remarkable ability to handle vast amounts of data, AI offers a versatile toolbox that extends its applications from diagnostic processes to the intricate world of surgical procedures.

What's exciting is that this isn't just a futuristic dream; it's happening now. AI algorithms actively decipher complex medical images, predict patient outcomes, and provide invaluable support to surgeons during complex procedures [1]. These incredible advancements are powered by the ever-growing availability of extensive medical data, ongoing refinement of machine learning algorithms, and a growing demand for healthcare solutions that are accurate and personalized to each patient.

Our motivation for this in-depth review arises from the rapid pace of progress in this field. While there's a wealth of literature on AI's integration into healthcare, there's a need for a comprehensive review that weaves together the various aspects of this emerging field. This review aims to provide a clear and broad view, shedding light on the potential benefits, challenges, and paths forward for AI's role in surgery and medicine [2].

Our objectives, though distinct, are closely intertwined. First, we would like to provide a thorough understanding of the current state of AI applications in both surgery and general medicine. This includes diving deep into AI's roles in diagnosing medical conditions, shaping treatment strategies, and assisting during surgeries. Secondly, our focus critically examines the complexities and ethical considerations of AI's integration into the healthcare system. We'll delve into the technical challenges and moral dilemmas that are part and parcel of this evolving landscape. Lastly, we endeavor to illuminate the potential future innovations and advancements that will shape the trajectory of AI within the medical field. We aim to project the horizons and the transformative potential that AI holds for healthcare.

This review will guide you through the current landscape of AI applications, starting with a detailed exploration of how AI contributes to diagnostics and treatment strategies. We'll then dive into the challenges, including technical complexities and ethical dilemmas. Finally, we'll look ahead to the future and explore the exciting possibilities AI brings to medicine [3].

Our review is designed to be both comprehensive and easy to understand, serving as a valuable resource for medical students and professionals. In today's era of digital medicine, the convergence of AI, surgery, and the broader field of medical practice promises a healthcare revolution. This revolution holds the potential to provide more precise diagnoses and more effective treatments, ultimately improving patient outcomes [4,5].

## Review

Historical context

Surgical medicine, an ancient practice dating back millennia, bears witness to an extraordinary evolution over the centuries. Its roots trace back to the earliest civilizations, where rudimentary tools and techniques were employed to address injuries and diseases [6]. From the ancient Sanskrit text, the "Sushruta Samhita," detailing surgical techniques in India, to the surgical arenas of ancient Rome, the journey of surgical medicine has been characterized by continuous learning, adaptation, and relentless innovation.

The 19th and 20th centuries marked a turning point in surgical medicine, ushering in groundbreaking advancements that revolutionized the field. Concepts like anesthesia, antiseptic techniques, and the introduction of advanced surgical instruments transformed the safety and efficacy of surgical procedures. These developments not only saved lives but also paved the way for more intricate and specialized surgical interventions [7].

In parallel, the latter half of the 20th century witnessed the dawn of AI. While the concept of AI, machines mimicking cognitive functions, was conceived early on, its practical applications in medicine took time to materialize [8]. Early AI systems in healthcare primarily consisted of rule-based expert systems designed to aid in diagnosis by following predefined algorithms.

The turn of the millennium marked a significant shift in the AI landscape, driven by the accessibility of large datasets and advancements in computational power. Deep learning, a subset of machine learning, emerged as a powerful tool for data analysis, finding applications from image recognition to natural language processing [4]. Within the medical field, AI began to demonstrate its potential in areas like radiology, where algorithms exhibited remarkable accuracy in detecting anomalies in medical images.

A pivotal milestone in the integration of AI into surgery was the development and approval of robotic surgical systems, including the renowned da Vinci Surgical System. These robotic systems, bolstered by AI capabilities, empowered surgeons to perform minimally invasive procedures with unprecedented precision and control [9]. Additionally, AI-driven predictive analytics played a vital role in patient monitoring, risk assessment, and postoperative care.

In recent years, the convergence of AI with surgical and general medicine has gained momentum. AI algorithms now assist in various aspects of healthcare, from patient triage to the customization of treatment plans. The integration of AI tools into electronic health records, telemedicine platforms, and wearable health devices is reshaping the patient-doctor relationship and the broader healthcare landscape, fostering a more patient-centric approach [10].

The historical journey of surgical medicine, intricately intertwined with the evolution of AI, stands as a testament to humanity's unceasing pursuit of knowledge and advancement. As we find ourselves at the crossroads of tradition and innovation, the future holds immense promise for further advancements in this symbiotic relationship. This historical context not only highlights the rich heritage of both surgical medicine and AI but also underscores the potential for groundbreaking developments on the horizon.

Theoretical foundations of AI

AI encompasses a multidisciplinary field with the overarching goal of creating machines that can replicate human intelligence. At its core, AI revolves around the development of algorithms that empower computers to perform tasks typically associated with human cognition [11]. To grasp the theoretical underpinnings of AI, it's essential to delve into its subfields, each with its unique contributions and applications.

Machine learning is a fundamental subset of AI and takes center stage in the quest for AI. Its primary focus lies in crafting algorithms that enable machines to learn from data and make informed decisions. Unlike traditional programming, which necessitates explicit instructions for every conceivable scenario, machine learning models harness the power of generalization. They adapt and evolve based on examples, becoming more adept at handling new data without requiring explicit reprogramming [12]. This adaptive capacity mirrors human learning, making it a cornerstone of AI's success.

Within the realm of machine learning, deep learning emerges as a formidable force. Deep learning models employ neural networks with numerous layers, earning them the moniker "deep" networks. These neural networks draw inspiration from the intricate structure of the human brain, featuring interconnected nodes or "neurons." The true strength of deep learning lies in its ability to process vast datasets and autonomously extract relevant features. This capacity has proven particularly impactful in tasks such as image and speech recognition, where deep learning algorithms have achieved remarkable accuracy by discerning patterns within immense pools of data [13].

In the ever-expanding landscape of AI, Natural Language Processing (NLP) stands as a pivotal domain. It centers on the interaction between computers and human language, a fundamental element of our communication. Within the realm of medical literature, NLP algorithms play a crucial role. They excel at extracting meaningful information from vast troves of unstructured text, facilitating a range of critical tasks. Automated medical coding, clinical decision support, and research data extraction are just a few examples of how NLP empowers AI to navigate and glean insights from the intricacies of human language in the medical domain [14].

Understanding these theoretical foundations of AI lays the groundwork for comprehending the transformative potential of AI within healthcare, a journey we will continue to explore in this review. As we delve deeper into AI's integration into medical diagnostics, surgical procedures, and therapeutics, keep these theoretical underpinnings in mind, as they underpin the remarkable advancements we're witnessing in the world of healthcare.

AI in medical diagnostics

The integration of AI into medical diagnostics is reshaping the landscape of healthcare. One of its most prominent domains of impact lies in the realm of medical imaging, where AI-driven techniques are ushering in a new era of precision and efficiency. From enhancing the interpretation of MRI and CT scans to aiding in real-time procedures, these algorithms are making a significant difference [15].

Beyond imaging, machine learning models are becoming invaluable allies in the diagnostic decision-making process. By harnessing the power of AI to analyze patient data, lab results, and historical cases, clinicians are gaining essential support in disease diagnosis, outcome prediction, and treatment recommendation [16].

The transformative potential of AI in diagnostics is underscored by compelling case studies. For instance, a study conducted at Stanford University showcased a deep learning algorithm's ability to detect skin cancer with accuracy on par with dermatologists [17]. Another study highlighted the use of AI in predicting cardiovascular risk through the analysis of retinal images, illustrating the promise of non-invasive diagnostic techniques [18].

AI in surgical procedures

The integration of AI into surgical procedures is ushering in a new era of unparalleled precision and efficiency within the operating room. One of the most groundbreaking applications is the realm of robotic surgery, exemplified by the da Vinci Surgical System. These robotic surgical systems have not only transformed but elevated minimally invasive procedures to new heights. With enhanced agility, stability, and high-definition magnification, they empower surgeons to perform procedures that are not only more precise but also less traumatic for patients [19].

However, the impact of AI extends far beyond the physical aspect of surgery. In the domain of surgical planning, AI algorithms are making significant strides by harnessing patient-specific data. These algorithms become invaluable partners to surgeons, aiding in preoperative planning, predicting potential complications, and even determining the optimal surgical approach. This not only reduces operative time but also translates into enhanced patient outcomes [20].

Moreover, AI is stepping into the realm of real-time decision-making support during surgery, marking yet another exciting frontier. In this scenario, algorithms provide surgeons with immediate, data-driven feedback, acting as invaluable guides as they navigate complex procedures. These AI-driven tools help identify critical structures, offer insights, and enable surgeons to make informed decisions on the fly, ultimately elevating the quality of care delivered within the operating room [20]. As AI continues to reshape the landscape of surgical procedures, it promises a future where precision and efficiency are no longer aspirations but everyday realities, benefitting both surgeons and their patients.

Advanced therapeutics

The field of therapeutics is undergoing a profound transformation fueled by the infusion of AI technologies. At the forefront of this revolution are nanobots, tiny robotic agents with the potential to revolutionize drug delivery. These minuscule marvels, programmable at the nanoscale, hold the promise of precisely targeting diseased cells with therapeutic agents. By doing so, they minimize side effects and enhance the efficacy of treatments, opening new horizons in patient care [21].

Additionally, AI is reshaping the landscape of personalized medicine, ushering in a new era of tailor-made treatments based on individual patient profiles. By harnessing vast datasets, including genomic information, AI algorithms now possess the capability to predict how patients will respond to various drugs, optimize dosages, and even identify potential drug candidates for rare and challenging diseases. This individualized approach ensures that patients receive treatments optimized for their unique genetic makeup, offering new hope for improved outcomes [22].

Moreover, AI's impact extends beyond the realm of treatment initiation. In the critical phase of postoperative care, AI algorithms are proving invaluable. They have the ability to monitor patient vital signs, predict potential complications, and optimize pain management strategies. This proactive approach ensures faster recovery times and shorter hospital stays, profoundly enhancing the overall patient experience [10]. As AI continues to pioneer advancements in therapeutics, the future promises treatments that are not only more precise but also increasingly tailored to the individual, revolutionizing healthcare delivery and improving patient outcomes.

Human-machine symbiosis

The integration of AI into healthcare heralds a new era, but it is not without its ethical and practical complexities. Ethical quandaries loom large, encompassing concerns such as patient consent, safeguarding data privacy, and the risk of algorithmic biases. It is imperative that AI tools adhere to the highest standards of patient care and safety [23].

The partnership between medical professionals and AI should not be misconstrued as a replacement; it is an augmentation. AI's proficiency in data processing and pattern recognition complements, rather than supplants, the irreplaceable qualities of the human touch, clinical intuition, and the nurturing of patient relationships. The ideal scenario envisions a symbiotic relationship where AI empowers healthcare providers [24].

Nevertheless, formidable challenges persist in the integration of AI tools. These hurdles encompass data interoperability issues, the necessity for rigorous validation processes, and the potential pitfall of excessive reliance on algorithms. Tackling these obstacles demands a multidisciplinary approach that unites technological innovation with clinical expertise [25].

By navigating these ethical and practical considerations, the healthcare landscape stands to gain immensely from the transformative potential of AI, ultimately leading to more effective, personalized, and patient-centric care.

The comprehensive impact

The integration of AI into surgical medicine and therapeutics carries profound implications across clinical, economic, and societal dimensions. On an economic front, the adoption of AI-driven tools and methodologies offers the promise of cost-effectiveness. By augmenting diagnostic precision, optimizing treatment strategies, and curbing hospitalization durations, AI stands to deliver substantial cost savings within healthcare systems. Nevertheless, it is essential to recognize that the initial investment in AI technologies and training can be substantial, necessitating a careful balance between upfront costs and long-term benefits for sustainable resource allocation [26].

Societally, the ascent of AI in medicine gives rise to a plethora of ethical considerations. Pressing issues such as data privacy, algorithmic transparency, and the potential biases inherent in AI models demand meticulous attention. Furthermore, the challenge of ensuring equitable access to AI-driven healthcare services, without exacerbating existing healthcare disparities, looms large on the societal horizon [27].

From the vantage point of patients, the ultimate gauge of success remains the quality of care. Preliminary findings offer promising indications that AI-enhanced procedures can lead to improved outcomes, diminished complications, and heightened patient satisfaction. The prospect of AI tailoring treatments to individual patients holds significant potential for enhancing overall quality of life [28].

In essence, the comprehensive impact of AI integration into surgical medicine and therapeutics extends far beyond mere technological innovation. It encompasses a profound transformation of healthcare dynamics, heralding a future marked by improved efficiency, enhanced equity, and, most importantly, superior patient care and well-being.

AI in patient engagement and education

The integration of AI into healthcare represents more than just a transformation of clinical procedures and diagnostics; it signifies a fundamental shift in the patient experience. As healthcare progressively embraces a patient-centric approach, AI plays a pivotal role in augmenting patient engagement and education, ensuring that individuals actively participate in their healthcare journey.

Patient Portals and AI-Driven Interfaces

Modern patient portals, harnessing the capabilities of AI, transcend their conventional roles as mere conduits for appointment bookings and test result retrieval. They have evolved into dynamic platforms that provide patients with personalized health insights, tailor-made advice, and proactive reminders. For example, an AI-driven patient portal can meticulously analyze a patient's medical history and lifestyle data to offer customized nutrition recommendations or exercise regimens [29].

Virtual Health Assistants

Virtual health assistants serve as formidable AI-driven tools in patient engagement, acting as round-the-clock health consultants. These digital companions guide patients through their healthcare journey, from pre-operative preparations to postoperative care. Virtual health assistants empower patients with autonomy by providing them with immediate access to a wealth of health-related information. They ensure that patients are well-informed at every step of their medical encounters, offering answers to queries, clarifying doubts, and providing reassurance whenever needed [30].

Educational Tools

AI is ushering in a revolution in health education, particularly when elucidating intricate medical procedures and patient treatment plans. AI-driven platforms generate various educational resources for individuals undergoing complex interventions, including easy-to-understand visualizations, interactive models, and even immersive virtual reality experiences. These resources serve as powerful aids in explaining the intricacies of a procedure, elucidating its potential benefits, and conveying associated risks comprehensibly. By making medical information more accessible and digestible, AI-driven educational tools empower patients to make informed decisions about their healthcare [31].

In the contemporary healthcare landscape, AI's role extends beyond clinical applications, penetrating the core of patient care. By enriching patient engagement and education, AI ensures that individuals are not passive recipients of healthcare but active participants in their well-being. This fosters a more holistic and patient-centered approach to medicine, where informed and engaged patients play a pivotal role in achieving better health outcomes.

Data security and privacy in AI-driven healthcare

The rapid integration of AI into healthcare brings with it an unprecedented surge in processed data. This exponential growth underscores the paramount importance of data security and the protection of patient privacy, which are two critical pillars upon which the ethical practice of AI in healthcare firmly rests.

Data Breaches and Their Implications

The healthcare sector, due to the sensitive and personal data it handles, stands as a prime target for cyberattacks. While AI-driven tools offer advanced diagnostics and treatment planning, they must be fortified with robust cybersecurity measures to prevent potential breaches. These breaches could have far-reaching consequences, not only in terms of patient trust but also incurring severe legal ramifications [32]. Ensuring the sanctity of patient data is a non-negotiable imperative, as it not only safeguards individuals but also upholds the integrity of the entire healthcare ecosystem.

Patient Consent and Data Usage

At the heart of ethical AI in healthcare lies transparency. Patients must be fully informed about how their data is utilized, especially if it's employed to train AI models. Obtaining explicit and informed patient consent and educating them about the use of their data is not just an ethical obligation but a fundamental building block for fostering trust [33]. It empowers individuals with agency over their health information, strengthening the patient-provider relationship.

Regulations and Compliance

In an era where data privacy is paramount, regulations such as GDPR in Europe and HIPAA in the United States play a pivotal role in safeguarding patient interests. Healthcare providers must uphold these regulations and ensure the highest data privacy standards. Given their inherently data-intensive operations, AI tools must be meticulously designed with these regulatory frameworks in mind. Compliance ensures legal adherence and, more importantly, underscores the commitment to safeguarding patient data and preserving their privacy [25].

The ethical practice of AI in healthcare demands unwavering dedication to data security and patient privacy. By fortifying cybersecurity measures, obtaining informed patient consent, and adhering to relevant regulations, the healthcare industry can harness the transformative potential of AI while ensuring the safety and trust of patients and the integrity of the healthcare ecosystem.

Training and skill development for medical professionals

The AI revolution in healthcare necessitates a parallel evolution in medical training and skill development.

Continuous Education

The medical curriculum must evolve to include AI and its applications in healthcare. Medical professionals should be equipped with the knowledge to leverage AI tools effectively and stay updated with the latest advancements [34]. Lifelong learning and continuous education programs are essential to ensure that healthcare practitioners remain proficient in the rapidly evolving AI landscape.

Simulation and Training Tools

AI-driven simulations, especially in surgical training, offer a risk-free environment for budding surgeons to practice and hone their skills. These simulations, often augmented with virtual or augmented reality, provide realistic scenarios, helping trainees prepare for real-world operations [35]. Such tools not only enhance skills but also improve patient safety by ensuring that surgeons are well-prepared for complex procedures.

Interdisciplinary Collaboration

The future of healthcare is collaborative. As AI tools become more intricate, their development and implementation require a joint effort from medical professionals, AI experts, and data scientists. Such interdisciplinary collaborations ensure that AI tools are clinically relevant, technically sound, and ethically developed [28]. By fostering collaboration, healthcare institutions can harness the full potential of AI while maintaining a focus on patient care and safety.

The integration of AI into healthcare is a transformative journey that demands continuous learning, practical training, and collaborative efforts. By embracing these principles, medical professionals can not only adapt to the AI-driven landscape but also contribute to its ethical and effective development for the benefit of patients and the healthcare industry as a whole.

Future directions

The horizon of AI in surgical medicine is vast and ever-evolving. Emerging technologies like quantum computing and augmented reality could further revolutionize the field, enabling even more sophisticated AI algorithms, real-time augmented surgical visualizations, and personalized patient simulations [34].

Predictions for the future are inherently speculative, but the trajectory suggests a more seamless integration of AI into all facets of medicine. We might witness AI-driven robots performing routine procedures autonomously, real-time AI analytics guiding complex surgeries, and AI-powered platforms for global collaboration and knowledge sharing [3]. This evolution represents a promising path toward more precise, efficient, and patient-centered healthcare practices.

Limitations of the review

Every review has its constraints, and this one is no exception. The scope of this review, while comprehensive, is partial. Specific niche applications of AI in surgical medicine might have yet to be covered in depth. The quality of existing literature varies. While many studies showcase the potential benefits of AI, rigorous, large-scale, and multi-center trials still need to be completed. This review has aimed to prioritize high-quality, peer-reviewed research, but study design and methodology variations exist.

Furthermore, there is a need for more extensive research in the current landscape. Many AI applications are in their infancy, and long-term outcomes, especially concerning rare complications or nuanced ethical dilemmas, might still need to be fully explored [36]. These limitations should be considered when interpreting the findings of this review. While it provides a comprehensive overview of AI's current role in surgical medicine, there is always room for further exploration and refinement as this exciting field continues to evolve.

## Conclusions

Integrating Artificial Intelligence (AI) with surgery and medicine is a momentous milestone in the ongoing evolution of healthcare. This comprehensive review meticulously traces the transformative journey of AI within the realms of diagnostics, surgical procedures, and therapeutics. We delve into the theoretical foundations of AI, highlighting its remarkable potential to augment and enhance clinical capabilities.

However, the path to incorporating AI into healthcare has its challenges. Ethical, economic, and societal considerations loom large, demanding careful navigation. As we peer into the future, it is imperative to acknowledge the limitations of current research and maintain a steadfast commitment to a balanced, patient-centric approach. Our compass should be guided by rigorous research and unwavering ethical principles.

This article reaffirms the pivotal role of AI in surgical medicine. We fervently hope that this review serves as a guiding beacon, advocating for the inclusion of AI in the medical literature. May it inspire and inform future endeavors, fostering progress that ultimately benefits patients and the broader healthcare landscape.
